# Totally endoscopic mitral valve repair and papillary muscle ablation for arrhythmic mitral regurgitation

**DOI:** 10.1186/s44215-025-00210-9

**Published:** 2025-05-28

**Authors:** Naoki Murata, Daisuke Endo, Hidemori Hayashi, Minoru Tabata

**Affiliations:** 1 Department of Cardiovascular Surgery, Juntendo University School of Medicine, 2-2-1, Hongo, Bunkyo, Tokyo, 113-8431 Japan; 2 Department of Cardiovascular Biology and Medicine, Juntendo University School of Medicine, 2-2-1, Hongo, 113-8431 Bunkyo, Tokyo, Japan

**Keywords:** Arrhythmic mitral regurgitation, Minimally invasive surgery, Cryoablation, Mitral regurgitation

## Abstract

**Background:**

Arrhythmic mitral regurgitation (MR), characterized by mitral valve prolapse accompanied by ventricular arrhythmias, poses diagnostic and therapeutic challenges. Treatment decisions, including the necessity for concomitant surgical ablation and the identification of specific ablation points, are highly case-specific and lack a consensus approach. Once determined, high-definition endoscopes and adaptable cryoablation tools facilitate simultaneous mitral valve repair and targeted surgical ablation via a minimally invasive approach, ensuring optimal visualization.

**Case presentation:**

The patient was a 49-year-old female with an episode of ventricular fibrillation. Severe MR was diagnosed at that time; however, surgery was not selected due to the absence of symptoms and left ventricular dysfunction. An implantable cardioverter-defibrillator was subsequently placed, which activated twice. Over the last 6 months, she developed dyspnea and was referred to us, where she was diagnosed with symptomatic arrhythmic MR. Subsequently, she underwent totally endoscopic minimally invasive mitral valve repair and papillary muscle cryoablation. Postoperative echocardiography showed trivial MR, with a mean pressure gradient of 1 mmHg, and an ejection fraction of 56%. She was discharged home on the fifth postoperative day. The Holter electrocardiogram performed 6 months after surgery showed no ventricular arrhythmias originating from the papillary muscles.

**Conclusions:**

We successfully diagnosed and treated a patient with symptomatic arrhythmic MR. Simultaneous mitral valve repair and papillary muscle cryoablation, performed through a totally endoscopic minimally invasive approach, was found to be effective in managing this complex condition.

## Background

Arrhythmic mitral regurgitation (MR) is defined as mitral valve prolapse accompanied by ventricular arrhythmias. There is currently no consensus on whether concomitant mitral valve surgery and ablation is effective as a treatment. Diagnostic and treatment strategies have not yet been established [1]. The cause of arrhythmia can be estimated by electrophysiological analysis, and in many cases, it has been reported to originate from fibrosis of the papillary muscles [2]. However, due to the complex anatomy of the papillary muscles, catheter ablation has a low success rate [1]. We report a case of symptomatic arrhythmic MR undergoing surgical mitral valve repair and papillary muscle ablation via a minimally invasive approach.

### Case presentation

The patient is a 49-year-old female who was referred to our institution for potential surgical treatment of severe mitral regurgitation. This condition was initially identified after she developed ventricular fibrillation (VF) and was resuscitated by a previous medical provider 3 years before. At that time, she was asymptomatic and elected not to undergo surgery for MR. She subsequently underwent implantation of a subcutaneous implantable cardioverter-defibrillator (ICD) at our institution, which activated twice during the follow-up period. Over the last 6 months, she began experiencing dyspnea on exertion, leading to the reconsideration of surgical intervention.

**Fig. 1:**
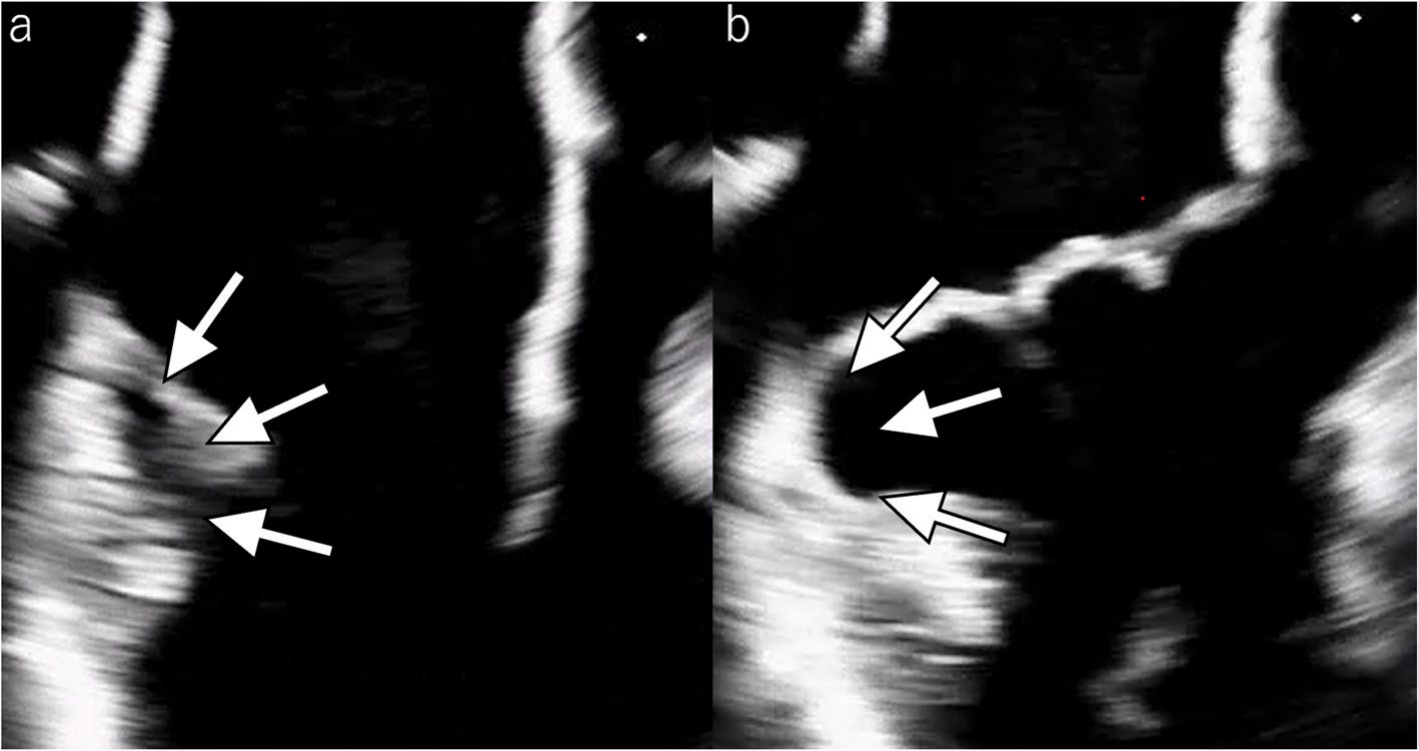
Preoperative transesophageal echocardiographic images (**a** diastole, **b** systole). The arrows indicate the areas of mitral annular disjunction

She had severe MR with preserved cardiac function and an ejection fraction of 62%. Transesophageal echocardiography showed degenerative change and prolapse of both mitral valve leaflets, without chordal rupture. There was marked annular dilatation and significant mitral annular disjunction on the posterior aspect (Fig. 1). The Pickelhaube sign characteristic of arrhythmic MR was absent.

**Fig. 2:**
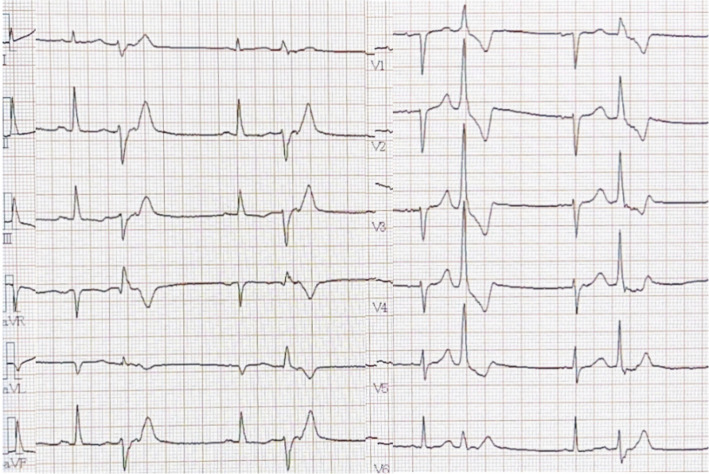
Preoperative premature ventricular contractions exhibited a right bundle branch block with left axis deviation, indicating an origin from the posterior papillary muscle

The electrocardiogram showed that frequent premature ventricular contractions (PVCs) had right bundle branch block (RBBB) with left axis deviation, suggesting a posterior papillary muscle origin (Fig. 2) The Holter electrocardiogram detected 2917 PVCs per day, accounting for 2.75% of all heartbeats. A relatively narrow QRS complex, suggestive of conduction through the His-Purkinje system rather than the ventricular myocardium, and a downward orientation of QRS waves in leads II, III, and aVF, indicating a possible origin from the posterior papillary muscle, were observed. However, due to limited evidence in the literature supporting the posterior papillary muscle as the arrhythmogenic site based on such criteria, the anterior papillary muscle was also targeted for treatment. Preoperative cardiac MRI was performed; however, due to the presence of an ICD device, the image quality was poor, making evaluation difficult.

**Fig. 3:**
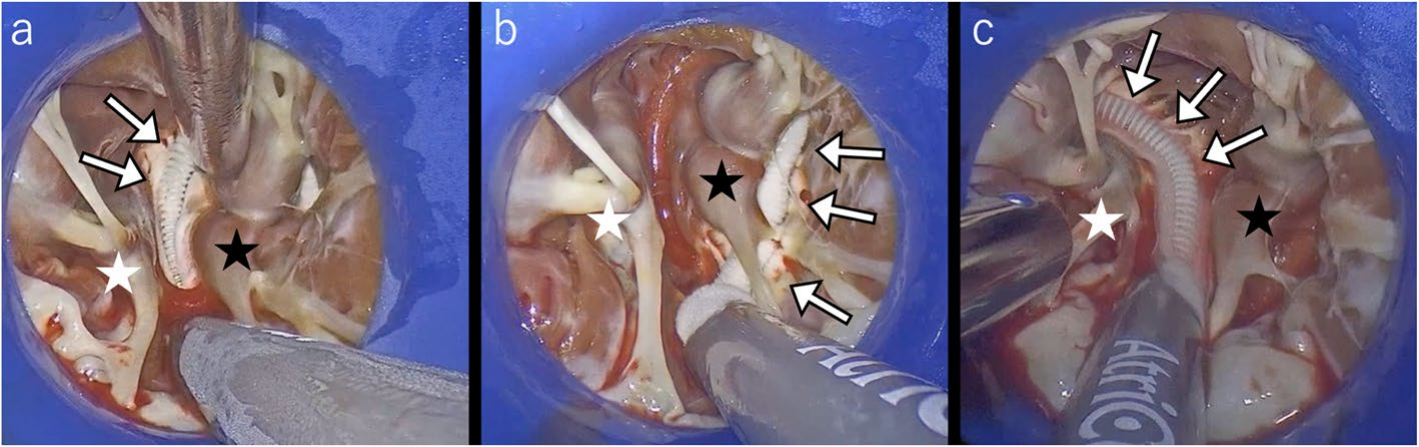
Intraoperative findings of papillary muscle cryoablation. The white star (☆) indicates the anterior papillary muscle, the black star (★) indicates the posterior papillary muscle, and the arrows indicate the cryoFORM probe

Simultaneous surgical mitral valve repair and cryoablation of the papillary muscles was performed through a totally endoscopic minimally invasive approach. A 3.5-cm skin incision was made, and a mini-thoracotomy was performed in the 4th intercostal space without the use of a rib spreader. A 4K 30-degree endoscope was inserted through a 5-mm port in the 5th intercostal space, while another port was placed in the 3rd intercostal space to accommodate left-hand instruments. A root cannula was inserted into the ascending aorta and connected to the myocardial protection circuit. Using a transthoracic aortic clamp, the ascending aorta was cross-clamped, and antegrade cardioplegia was initiated. Through a left atriotomy, the mitral valve was exposed. Both leaflets of the mitral valve showed prolapse with significant myxomatous changes of the leaflets and chordae tendineae. Then, the left ventricle was exposed with a ring-type retractor, ensuring excellent visualization of the papillary muscles with a 4K endoscopy. For exposure of the subvalvular structures, we used the ring-shaped MitroB Heart Valve Retractor (LANDANGER, France), which safely retracted the leaflets and chordae, allowing clear exposure of the papillary muscle. For cryoablation, the cryoFORM device (AtriCure, Innovation Way, Mason, OH, USA) was used to target the bases of both papillary muscles, further enhanced by the 4K endoscopy for precision (Fig. 3). Although circumferential ablation at the anterior papillary muscle base was planned, we ablated only the medial side because the lateral side merged with the ventricle. Mitral valve repair was then performed. The procedure involved the placement of six pairs of artificial chords (two on the anterior leaflet, three on the posterior leaflet, and one on the posterior commissure), closure of the indentation between P2 and P3 scallops, and annuloplasty with a 36-mm full semirigid ring. The total duration of surgery, cardiopulmonary bypass, and cross-clamp was 252, 173, and 133 min, respectively.

Postoperative transthoracic echocardiography showed trivial MR with a mean pressure gradient of 1 mmHg. The patient was uneventfully discharged home on the fifth postoperative day. Regarding postoperative pharmacological treatment, the preoperative bisoprolol 2.5 mg was continued. IV amiodarone was initiated intraoperatively and switched to oral amiodarone 200 mg qid postoperatively. It was reduced to 100 mg at 1 month and discontinued at 3 months.

The Holter electrocardiogram performed 6 months postoperatively showed PVCs occurring at a rate of 0.18%, representing a 93% reduction compared to preoperative levels. Importantly, none of the residual PVCs originated from the papillary muscles, suggesting a curative effect of the ablation on papillary muscle-triggered PVCs. No recurrences of severe ventricular arrhythmias, such as VF, were observed.

## Discussion

The key points of this case are as follows: We successfully eliminated the papillary muscle-derived PVC by performing mitral valve repair and papillary muscle ablation. The use of an endoscope provided an excellent visualization of the left ventricle as well as the mitral valve. The cryoFORM was particularly useful for ablation within the narrow confines of the left ventricle.

Arrhythmic MR is defined as a condition where ventricular arrhythmias coexist with MR in the absence of other organic changes that could cause arrhythmias [3]. The prevalence of arrhythmic MR and its potential genetic predisposition remain unclear [4, 5]. The primary pathology involves conduction system abnormalities due to myocardial degeneration. Mitral valve prolapse causes nonelastic fibrous chordae to pull papillary muscles toward the left atrium during systole, leading to degeneration. This degeneration affects Purkinje fibers within the papillary muscles, potentially inducing arrhythmias [6, 7]. Arrhythmic mitral regurgitation (AMR) is hypothesized to result from papillary muscle degeneration via the chordae, secondary to mitral annulus enlargement (“MR-induced arrhythmia”). AMR refers to patients with mitral valve prolapse and frequent or complex ventricular arrhythmias, without other arrhythmic substrates. Its management and prognosis remain unclear, highlighting the need for improved risk prediction, diagnosis, and treatment [3].

Diagnosis of arrhythmic MR starts with confirming mitral regurgitation and detecting ventricular arrhythmias like PVC, VT, or VF using echocardiography and a 12-lead ECG or electrophysiological study (EPS). PVCs often originate from the posterior papillary muscle, and when they arise from the left posterior fascicle of the Purkinje fibers, the ECG typically shows RBBB with left axis deviation [2]. The Pickelhaube sign on echocardiography, indicative of stress on the mitral apparatus, suggests significant fibrosis and conduction abnormalities [8].

Treatment involves surgical intervention for severe MR and simultaneous cryoablation, though long-term outcomes remain unclear [2, 9]. In our case, however, the cardiology team at the time did not choose surgery. It is presumed that the concept of arrhythmic MR was not yet well established, the patient had no heart failure symptoms, and the degenerative MR was considered too complex for a durable repair. Based on more recent knowledge, it appears that surgery would have been indicated at the time of the ventricular fibrillation episode. Catheter ablation is challenging due to the complex and narrow left ventricular cavity, particularly around the base of the papillary muscles where many trabeculae are present, making it difficult to maintain stable catheter contact [10]. Postoperative ICD implantation is recommended to prevent sudden death [11].

The advantages of surgical treatment include the ability to perform mitral valve repair and ablation simultaneously, excellent direct or endoscopic visualization of the papillary muscles as well as the mitral valve, and the ease of complex papillary muscle ablation using a slim flexible cryoprobe.

We believe that the PVCs in our case were eliminated through two key mechanisms. First, controlling MR via MVR reduced the stress on the papillary muscles by preventing the mitral valve leaflets from being pulled toward the left atrium, thereby decreasing arrhythmia induction. Although the case involved polymorphic PVCs, isolated MVR can be effective in preventing PVC recurrence, especially when preoperative PVCs are of a low Lown grade (grade 2 or less) [12].

Second, cryoablation on the papillary muscles was effective [13]. Unlike radiofrequency ablation, which often fails due to unstable contact with the papillary muscles, cryoablation provides stable contact and induces tissue necrosis, leading to favorable outcomes without significant complications [14, 15]. Given these points, performing cryoablation under direct or endoscopic vision during MVR is a rational approach.

A key issue in ablation is maintaining stable probe contact for effective energy delivery. High-definition endoscopy in a minimally invasive approach enhances this process and maneuverability by offering a clearer view of the left ventricle, overcoming the limitations of direct visualization through the mitral or aortic valve [16, 17].

Lastly, cryoFORM is particularly effective for ablation in the narrow space of the left ventricle, where the complex anatomy of the papillary muscles makes accurate ablation challenging with conventional catheter-based treatments. CryoFORM induces cryonecrosis by freezing the target tissue, blocking electrical conduction pathways. Its flexible probe adapts well to the tight spaces within the left ventricle, and its ergonomically designed handle improves operability [18].

In conclusion, this case demonstrates the successful elimination of papillary muscle-derived PVCs through mitral valve repair and cryoablation, facilitated by endoscopic visualization. The use of cryoFORM in the narrow left ventricle proved effective, significantly reducing arrhythmias. These findings suggest that surgical intervention, combined with advanced endoscopic techniques, can be a valuable approach for managing arrhythmic MR.

## Conclusions

We successfully diagnosed and treated a patient with symptomatic arrhythmic MR. Simultaneous mitral valve repair and papillary muscle cryoablation, performed through a totally endoscopic minimally invasive approach, with the use of a 4 K endoscope and slim, flexible cryoprobe was found to be effective in managing this complex condition. This approach highlights the benefits of advanced surgical techniques in treating arrhythmic MR, providing a foundation for similar future interventions.

## Data Availability

Not applicable.

## Abbreviations

MR: Mitral regurgitation
VF: Ventricular fibrillation
ICD: Implantable cardioverter-defibrillator
MVR: Mitral valve repair
PVCs: Premature ventricular contractions

## References

[1] Turnbull S, Kumar S, Campbell T. Surgical cryoablation of malignant papillary muscle arrhythmias during mitral valve prolapse surgery - putting a freeze on sudden cardiac death. Heart Lung Circ. 2022;31:1318–20. 36162874 10.1016/j.hlc.2022.08.005

[2] El-Eshmawi A, Pandis D, Miller MA, Boateng P, Dukkipati SR, Reddy VY, et al. Surgical cryoablation of papillary muscle PVCs during mitral valve surgery: therapeutic consideration for malignant MVP. J Am Coll Cardiol. 2020;76:3061–2. 33334428 10.1016/j.jacc.2020.10.037

[3] Kubala M, Essayagh B, Michelena HI, Enriquez-Sarano M, Tribouilloy C. Arrhythmic mitral valve prolapse in 2023: evidence-based update. Front Cardiovasc Med. 2023;10:1130174. 10.3389/fcvm.2023.1130174. 10.3389/fcvm.2023.1130174 PMC10153002 37144062

[4] Duren DR, Becker AE, Dunning AJ. Long-term follow-up of idiopathic mitral valve prolapse in 300 patients: a prospective study. J Am Coll Cardiol. 1988;11:42–7. 10.1016/0735-1097(88)90164-7. 10.1016/0735-1097(88)90164-7 3335704

[5] Grigioni F, Enriquez-Sarano M, Ling LH, Bailey KR, Seward JB, Tajik AJ, et al. Sudden death in mitral regurgitation due to flail leaflet. J Am Coll Cardiol. 1999. 10.1016/s0735-1097(99)00474-x. 10588227 10.1016/s0735-1097(99)00474-x

[6] Nishimura RA, McGoon MD, Shub C, Miller Jr FA, Ilstrup DM, Tajik AJ. Echocardiographically documented mitral-valve prolapse. Long-term follow-up of 237 patients. N Engl J Med. 1985;313:1305–9. 10.1056/NEJM198511213132101. 10.1056/NEJM198511213132101 4058522

[7] El Sabbagh A, Reddy YNV, Nishimura RA. Mitral valve regurgitation in the contemporary era: insights into diagnosis, management, and future directions. JACC Cardiovasc Imaging. 2018. 10.1016/j.jcmg.2018.01.009. 29622181 10.1016/j.jcmg.2018.01.009

[8] Lakshmi M, Faraaz R, Fuad J, Shaikh A, Kalvin L, Dhala A, et al. The Pickelhaube sign: novel echocardiographic risk marker for malignant mitral valve prolapse syndrome. JACC Cardiovasc Imaging. 2017. 10.1016/j.jcmg.2016.09.016. 10.1016/j.jcmg.2016.09.016 28017396

[9] Van DPF, Hemel NMV, Swieten HAV, Bakker JMD, Jessurun ER. Successful surgical ablation of sustained ventricular tachycardia associated with mitral valve prolapse guided by a multielectrode basket catheter. Pacing Clin Electrophysiol: PACE. 2001. 10.1046/j.1460-9592.2001.01029.x. 10.1046/j.1460-9592.2001.01029.x 11449580

[10] Seiler J, Lee JC, Roberts-Thomson KC, Stevenson WG, et al. Intracardiac echocardiography guided catheter ablation of incessant ventricular tachycardia from the posterior papillary muscle causing tachycardia mediated cardiomyopathy. Heart Rhythm. 2009. 10.1016/j.hrthm.2008.11.029. 19251217 10.1016/j.hrthm.2008.11.029

[11] Priori SG, Blomstrom-Lundqvist C, Mazzanti A, Blom N, Borggrefe M, Camm J, et al. 2015 ESC guidelines for the management of patients with ventricular arrhythmias and the prevention of sudden cardiac death: the task force for the management of patients with ventricular arrhythmias and the prevention of sudden cardiac death of the European Society of Cardiology (ESC). endorsed by: association for European paediatric and congenital cardiology (AEPC). Eur Heart J. 2015;41:2793–867. 10.1093/eurheartj/ehv316. 10.1093/eurheartj/ehv316 26320108

[12] Pandis D, David N, El-Eshmawi A, Miller MA, Boateng P, Costa AC, et al. Noncomplex ventricular arrhythmia associated with greater freedom from recurrent ectopy at 1 year after mitral repair surgery. JTCVS Open. 2024. 10.1016/j.xjon.2024.04.005. 39015439 10.1016/j.xjon.2024.04.005 PMC11247206

[13] Kautzner J, Peichl P. Papillary muscle ventricular tachycardia or ectopy: diagnostics, catheter ablation and the role of intracardiac echocardiography. Arrhythm Electrophysiol Rev. 2019. 10.15420/aer.2018.80.2. 30918670 10.15420/aer.2018.80.2 PMC6434512

[14] Rivera S, Ricapito M, Espinoza J, Belardi D, Albina G, Giniger A, et al. Cryoablation for ventricular arrhythmias arising from the papillary muscles of the left ventricle guided by intracardiac echocardiography and image integration. Clin Electrophysiol. 2015. 10.1016/j.jacep.2015.07.012. 10.1016/j.jacep.2015.07.012 29759404

[15] Gordon JP, Liang JJ, Pathak RK, Zado ES, Garcia FC, Hutchinson MD, et al. Percutaneous cryoablation for papillary muscle ventricular arrhythmias after failed radiofrequency catheter ablation. J Cardiovasc Electrophysiol. 2018. 10.1111/jce.13716. 30106213 10.1111/jce.13716

[16] Misumi T, Kudo M, Koizumi K, Yamazaki M, Nakagawa M, Kumamaru H. Intraoperative endoscopic resection of left ventricular tumors. Surg Today. 2005. 10.1007/s00595-004-3086-9. 16341495 10.1007/s00595-004-3086-9

[17] Burns DJP, Wierup P, Gillinov MDaniel JPB. Minimally invasive mitral surgery: patient selection and technique. Cardiol Clin. 2021;39:211–20. 33894935 10.1016/j.ccl.2021.01.003

[18] Schroeter T, Misfeld MThomas S. Characteristics of the new AtriCure cryoFORM® cryoablation probe for the surgical treatment of cardiac arrhythmias. Expert Rec Med Devices. 2017;14:255–62. 10.1080/17434440.2017.1309972 28326843

